# The corpulent phenotype—how the brain maximizes survival in stressful environments

**DOI:** 10.3389/fnins.2013.00047

**Published:** 2013-04-02

**Authors:** Achim Peters, Britta Kubera, Christian Hubold, Dirk Langemann

**Affiliations:** ^1^Clinical Research Group: Brain Metabolism, Neuroenergetics, Obesity and Diabetes, University of LuebeckLuebeck, Germany; ^2^Computational Mathematics, Technical University of BraunschweigBraunschweig, Germany

**Keywords:** mortality, obesity, phenotypic plasticity, selfish brain theory, stress reactivity

## Abstract

The reactivity of the stress system may change during the life course. In many—but not all—humans the stress reactivity decreases, once the individual is chronically exposed to a stressful and unsafe environment (e.g., poverty, work with high demands, unhappy martial relationship). Such an adaptation is referred to as habituation. Stress habituation allows alleviating the burden of chronic stress, particularly cardiovascular morbidity and mortality. Interestingly, two recent experiments demonstrated low stress reactivity during a mental or psychosocial challenge in subjects with a high body mass. In this focused review we attempt to integrate these experimental findings in a larger context. Are these data compatible with data sets showing a prolonged life expectancy in corpulent people? From the perspective of neuroenergetics, we here raise the question whether “obesity” is unhealthy at all. Is the corpulent phenotype possibly the result of “adaptive phenotypic plasticity” allowing optimized survival in stressful environments?

## Introduction

“Obesity” is commonly regarded as a disease. The term is derived from the Latin *obesus “having eaten until fat”* (from *ob- “away, completely” + esus* past participle of *edere “eat”*). As this word origin suggests, “obesity” is widely considered as a controllable condition, and its cause is often attributed to a lack of willpower or discipline (Sikorski et al., 2012). Recent clinical data, however, clearly contradict the prejudice of weak will, since children and adults with high body mass index (BMI) display a stronger cognitive control over eating behavior than all others (Timko and Perone, 2005; de Lauzon-Guillain et al., 2006; Snoek et al., 2008; Gallant et al., 2010). Moreover, with upcoming new epidemiological data the question arises whether a high BMI is unhealthy at all (Flegal et al., 2013).

The nephrologists were the first who reported that patients on dialysis treatment had better survival chances when they had a higher BMI (Kopple et al., 1999). This phenomenon—often referred to as the “**obesity paradox**”, has also been observed in other diseases like myocardial infarction, stroke, pulmonary disease, sepsis, and type 2 diabetes mellitus (Buettner et al., 2007; Hallin et al., 2007; Vemmos et al., 2011; Carnethon et al., 2012). In the first place, these data sets had been classified as special cases being only relevant in intensive care units. However, recent studies in general populations from Mauritius, Denmark, and Great Britain showed that people with higher BMI (“overweight” and “obese”) live longer than those with lower BMI (“normal weight”) (Berentzen et al., 2010; Cameron et al., 2012; Hamer and Stamatakis, 2012). These observations raised doubt about the common assumption that obesity is a harmful disease. Is it even possible that in a stressful environment a “corpulent phenotype” develops which is more stress tolerant and finally has better survival chances?

Here we argue from a perspective of neuroenergetics and we shall provide evidence supporting the notion that weight gain in humans is an adaptive strategy allowing better survival in stressful-unsafe environments.

## The selfish brain

**Figure 1:**
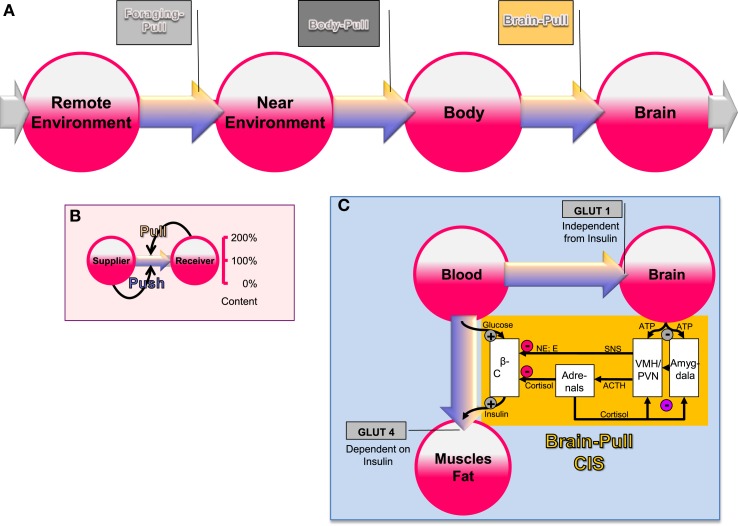
**General supply chain of the human brain. (A)** Overview: energy from the remote environment is brought to the immediate environment, the body then takes it up, and from there a large part of it enters the brain. **(B)** The push-pull principle: in a general supply chain, the flux of energy can principally be determined by the supplier (previous step) or by the receiver (proximate step). The share of the flux, which is determined by the supplier, is called the “push component” (blue arrows), the share, which is determined by the receiver, is called the “pull component” (yellow arrows). **(C)** Conceptual diagram that shows key physiological mechanisms, which fulfill the brain-pull function of cerebral insulin suppression (CIS): the flux of glucose is either directed to the brain or to muscle and fat. If neuronal ATP concentrations fall, activation of neurons in the amygdala, in the ventromedial hypothalamus (VMH), and in the paraventricular nucleus (PVN) occurs, which in turn activates the sympathetic nervous system and the hypothalamus pituitary adrenal system (SNS and HPA). Both the SNS and the HPA system suppress insulin release from pancreatic beta cells, thereby decreasing glucose transporter 4 (GLUT-4) mediated glucose uptake into muscle and fat. In this way the brain limits glucose uptake in peripheral tissues and favors glucose transporter 1 (GLUT-1) mediated glucose uptake into the brain. Finally, the CIS-brain-pull system is hierarchically organized and adaptive. The central glucocorticoid feedback plays a critical role in determining whether the CIS-brain-pull system reacts in a high or low reactive manner. ACTH, Adrenocorticotropic hormone; E, epinephrine; NE, norepinephrine.

The brain occupies a special hierarchical position in human energy metabolism (Peters et al., 2004). Almost 100 years ago Marie Krieger provided first scientific evidence for the central position of the brain in energy metabolism. She reported that in the state of inanition all the organs like the heart, the liver, and the kidney lost approximately 40% of their mass, while the brain mass hardly changed (less than 2%) (Krieger, 1921). Using a mathematical logistic approach, which is based on the principles of “supply and demand” known from economics, we could show that brain supply must be regulated both by *offer* and *demand* (Peters and Langemann, 2009). In order to show this, we have designed a brain-supply-chain model (Figure 1A). Because the brain has been demonstrated to be both the principle consumer *and* the principle controller in energy metabolism, we have regarded it as the final consumer in this model. Energy from the remote environment is brought to the immediate environment, then the body takes it up (into the blood stream), and from there approximately 2/3 of the circulating glucose enters in the brain. Supply chains in industrial production processes display striking similarities to the glucose pathway from the environment through the body toward the brain. In the field of logistics such supply chains have been extensively studied. Over the decades, a number of basic principles have been formulated and elaborated (Slack et al., 2004). The so-called “push”-principle operates according to the following rule (Figure 1B): the supplier delivers material and in so doing determines the activity of a production step. In contrast, the so-called “pull”-principle works in the following manner: the material required for a production step is provided only when the receiver needs it (just-in-time). In comparison with the “push”-principle the “pull”-principle offers clear economic advantages; with the latter there are short set-up times and only small (economically optimized) storage sites. Many modern industrial branches have recognized that pull components are particularly efficient. While designing our brain-supply-chain model we referred to the basic principles of general supply chains. With the help of such a brain-supply-chain model we could show that the existence of an active cerebral-demand process is indispensable for explaining the observed brain mass preservation, which occurs under conditions of food deprivation. The force with which the brain actively demands energy from the body is referred to as “**brain-pull**.”

First evidence for the existence of brain-pull mechanisms came from the laboratory studies of Luc Pellerin and Pierre Magistretti (Magistretti et al., 1999). On the cell-to-cell level, the researchers showed that neurons actively demand energy from the blood via astrocytes. On the systemic level, brain-pull mechanisms have been discovered, which allow allocation of energy from the body toward the brain. One key brain-pull mechanism is referred to as “**cerebral insulin suppression” (CIS)** (Kubera et al., 2012). CIS operates in following way (Figure 1C): both the medial amygdala nucleus and the ventromedial hypothalamus contain glucose-responsive neurons, which control the activity of the sympathetic nervous system (SNS) and the hypothalamus-pituitary adrenal axis (HPA) (Miki et al., 2001; Zhou et al., 2010). The medial amygdala nucleus sends heavy input to the ventromedial hypothalamus and thereby exerts control over its activity (Petrovich et al., 2001). In the ventromedial hypothalamus, ATP-sensitive potassium (KATP) channels have been shown to monitor intracellular ATP (Spanswick et al., 1997). If the cerebral intracellular ATP concentrations fall, ventromedial hypothalamus neurons depolarize due to GABAergic disinhibition (Chan et al., 2007). Via glutamatergic mechanisms they activate the sympathoadrenal system (Tong et al., 2007). Specific sympathetic efferences project to pancreatic beta-cells and in so doing inhibit insulin secretion (Ahren, 2000). Ventromedial hypothalamus-controlled sympathetic efferences also project to muscle and fat tissue, where they inhibit insulin-dependent glucose transport via GLUT 4 (Mulder et al., 2005). Thus, the ventromedial hypothalamus neurons are capable of limiting the flux of glucose from the blood into the peripheral energy stores (muscle and fat tissue). As a consequence, glucose is now available via insulin-independent GLUT1-transport across the blood-brain barrier (Hasselbalch et al., 1999; Seaquist et al., 2001). CIS can be interpreted as a brain-pull mechanism that functions to demand energy from the body. In this way, the brain is able to adjust its energy supply to its varying energy needs.

Cerebral energy demand mechanisms like CIS are activated during psychosocial stress. During *acute* stress episodes, vigilance increases; the reaction time decreases, and alternative behavioral strategies are searched for (exploration). This stress-induced behavioral state of hypervigilance requires extra energy (Madsen et al., 1995; Hitze et al., 2010). By activating brain-pull mechanisms the brain demands for extra energy from the body and thereby covers such cerebral extra needs. The stress-induced behavioral state allows high cerebral functioning for problem solving—but this special functional mode costs additional energy. In all, a high-reactive stress system is required for entering a highly functional cerebral mode during stress.

## Habituation of the stress response

**Figure 2:**
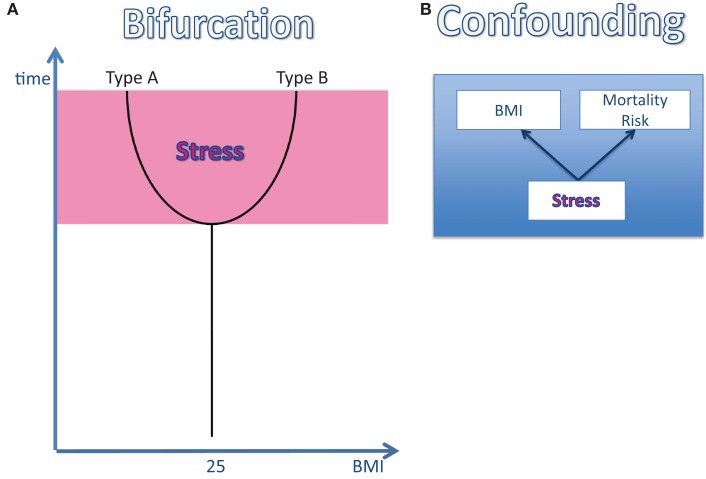
**The effect of stress on body weight and mortality risk. (A)** The bifurcation of the life course: once an individual is exposed to a stressful environment its body weight changes: in type-A-individuals the body mass index (BMI) decreases, while in type-B the BMI increases. **(B)** The confounding factor “stress”: stress has been shown to affect both the BMI and the mortality risk. Therefore, stress can be regarded as a confounding factor, which definitely influences the BMI-mortality association.

The reactivity of the stress system may change during the life course. Once exposed to a stressful environment (e.g., poverty, unhappy martial relationship, high demand at work, loneliness), humans are known to react in two distinct patterns (Kirschbaum et al., 1995): type-A-individuals are characterized by their maintenance of a high-reactive stress response under conditions of permanent or repeated stress. They do not show any signs of habituation. And they lose body weight (Figure 2A). In contrast, type-B-individuals, who initially responded to stress in a high-reactive manner, decrease their stress response by time and in this way they continue to respond to stress in a low-reactive manner. They do exhibit signs of habituation. And they gain weight (Figure 2A). As estimated from data in a young study population from Great Britain, 50% of the participating individuals can be considered as type A and 50% as type B (Oliver and Wardle, 1999).

The molecular basis underlying the process of stress habituation has recently been identified. The endocannabinoid system in the prefrontal cortex plays a key role in stress habituation (Hill et al., 2010). If stressors cannot be defended successfully in the long run, then a learning process involving synaptic plasticity (long-term potentiation; LTP) within the prefrontal cortex allows suppressing the potential amygdala response to the stressor (Freund et al., 2003; Hill and Tasker, 2012). The induction of LTP at synapses is facilitated by the endocannabinoid system (Carlson et al., 2002). The synaptic changes during stress habituation result in a damped response of the SNS and the hypothalamus-pituitary-adrenal axis (Kirschbaum et al., 1995). Whether an individual exhibits a type-A- or a type-B-stress response is determined by the characteristics of the endocannabinoid systems. These characteristics are essentially based on genetic factors. Thus, the genetic predisposition determines whether signs of habituation are present or absent when the individual is exposed to chronic stress.

Evolutionary biology has extensively studied adaptations to environmental changes. When environments within the range of a species differ, it is unlikely that any single phenotype will confer high fitness in all situations. In such a case, a change in the phenotype (e.g., change in the stress reactivity or interchange between asexual and sexual reproduction) that depends on the environment (**phenotypic plasticity**) can provide increased environmental tolerance (Via et al., 1995; Agrawal, 2001).

## The benefits and costs of a low-reactive stress system

In 1993 Bruce McEwen introduced the concept of *allostatic load* into medicine (McEwen and Stellar, 1993). **Allostatic load** refers to the damaging effect of a chronically activated stress response. It has been shown that chronically stressed individuals do not only display high levels of serum cortisol and weight loss, but are also at high risk to die earlier, become infertile, develop arteriosclerosis, arterial hypertension, coronary heart disease, myocardial infarction, stroke, typical depression, muscle atrophy, and osteoporosis. In principle, a high-reactive stress system may be highly effective in defending against stressors. But if removal of the stressor or escape from it is impossible, then a permanent high-reactive stress system turns out to be detrimental.

Therefore, habituation of the stress response is of benefit, if the stressor cannot be successfully removed. Then, habituation reduces the damaging effects of the allostatic load. Habituation results in a maintained biological fitness once the individual is set into a stressful-insecure environment.

On the other hand, habituation of the stress response is also costly. Once habituation occurs in the stress system, the brain-pull function—usually carried out by a high-reactive stress system—is no longer effective. Then, the brain is unable to demand for sufficient energy from the body. There is, however, one alternative to preserve cerebral energy homeostasis under these adverse circumstances: the increase of ingestive behavior. If the food offer is abundant, then individuals who have developed a low-reactive stress system are able to procure their brain by a compensatory increase in food intake. However, this alternative strategy of the brain has an unwanted side effect: weight gain. Using the logistic model of the **cerebral supply chain**, it has been proven analytically that the reactivity of the brain-pull function is strictly inverse to the energy fill level of the body (body mass) (Peters and Langemann, 2009). In other words: a high-reactive stress system allows effectively demanding energy from body stores; it guarantees immediate adjustment of the brain's energy supply to its varying energy needs. In contrast, a low-reactive stress system does not allow sufficiently procuring energy from body stores; as a compensatory strategy, the brain may switch from brain-pull to **body-pull** (Figure 1A); in this way, the brain's needs are covered, but the surplus of energy accumulates in the body—and the result is weight gain. Thus, we may consider weight gain as the cost, which has to be paid for the adaptive benefits of stress habituation.

In summary, stress habituation can be regarded as an adaptive and beneficial process, which reduces the allostatic load under chronic stress conditions; and it also is associated with the costs of weight gain.

## Low-reactive stress system in the corpulent phenotype

Long-term observational studies have demonstrated that a low-reactivity of the stress system—either measured by the adrenalin response during a mental stress test or by heart rate variability—is a strong predictor of weight gain (Carroll et al., 2008; Flaa et al., 2008). Twenty-five years ago, it has already been recognized that body fat is related to a low-reactivity of the autonomic nervous system (Peterson et al., 1988). The higher the body fat was, the smaller the cardiac responses to stress were, the lower the catecholamine concentrations and the pupillary reactions. At that time, it has already been assumed that the reduced stress system's reactivity plays a causal role in weight gain.

**Figure 3:**
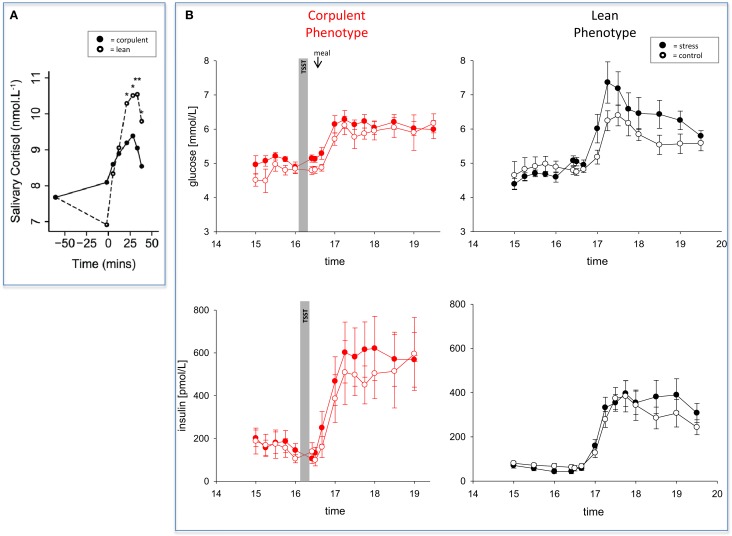
**The effect of mental stress on cortisol and CIS in lean and corpulent humans. (A)** HPA-reactivity is low in corpulent subjects: lean subjects respond to a mental stressor in a high-reactive manner, while corpulent subjects respond to it in a low-reactive manner (Jones et al., 2012). **(B)** CIS is not detectable in corpulent subjects: glucose and insulin concentrations are measured during stress and non-stress intervention in corpulent and lean men. Values are means ± SEM; closed symbols, stress intervention and open symbols, non-stress intervention. Lean men: glucose concentrations are different from non-stress intervention (interaction time × stress intervention: *F* = 3.8, *P* < 0.001); insulin concentrations are equal between stress and non-stress intervention (interaction time × stress intervention: *F* = 2.5, *P* > 0.05), by ANOVA for repeated measures. Given the increased glucose concentrations in the stress session, insulin is inadequately low, i.e. suppressed (for details see Hitze et al., 2010). Corpulent men: stress did not lead to an observable increase in glucose concentrations (interaction × stress intervention: *F* = 0.6, *P* = 0.554) nor in insulin concentrations in the post-stress replenishment phase (interaction time × stress intervention: *F* = 0.4, *P* = 0.771) (Kubera et al., 2012).

In 2012, two papers have been published almost simultaneously both confirming that women and men with high BMI do react to stress in a low-reactive manner (Jones et al., 2012; Kubera et al., 2012). The two research groups applied a mental and a psychosocial stress test, respectively. They found blunted neuroendocrine, neuroenergetic, emotional, and cardiovascular responses to stress (Table 1). As a hallmark, both studies found a low-reactivity of the hypothalamus-pituitary-adrenal axis in people with high BMI (Figure 3A). One of these two studies came from our lab and we shall report here the results in greater details.

**Table 1:** **Differential stress reactivity in lean and corpulent subjects**.

**Stress reactivity**		**Lean subjects (BMI 18–25)**	**Corpulent subjects (BMI > 30)**	**Evidence**
Neuroendocrine	Cortisol to acute mental stress	High	Low	Jones et al., 2012
	Cortisol to subsequent meal	High	Low	Kubera et al., 2012
Neuroenergetic	Hypervigilant state	High	Low	Kubera et al., 2012
	Cerebral insulin suppression (CIS)	High	Low	Kubera et al., 2012
	Poststress neuroglycopenic state	High	Low	Kubera et al., 2012
Emotional	Anxiety, uneasiness, physical malaise, sadness	High	Low	Kubera et al., 2012
Cardiovascular	Heart rate, blood pressure, cardiac output	High	Low	Jones et al., 2012

## The corpulent phenotype exhibits a robust brain metabolism during stress

To investigate how the brain of lean or corpulent humans organizes its supply and demand during psychosocial stress, we performed the Trier Social Stress Test in 40 men, and thereafter offered them a rich food buffet (Kubera et al., 2012). Lean men were found high-reactive during the stress session; they increased their vigilance; thereby they increased their cerebral energy need. In contrast, corpulent men were found low reactive in this respect. Neither a hypervigilant nor a neuroglycopenic state was detectable in the corpulent subjects (Table 1).

Whereas the brains of normal-weight subjects demanded for extra energy from the body using CIS, CIS was not detectable in corpulent subjects (Figure 3B) (Kubera et al., 2012). Our findings suggest that the absence of CIS in corpulent subjects was due to the absence of their meal-related-cortisol peak. That means that in corpulent men brain-pull function was not sufficiently activated to demand for energy to procure the brain. Our results indicate that in lean subjects, who highly activated their CIS-brain-pull, the energy in the post-stress-replenishment phase was rather directed toward the brain. In contrast, the corpulent subjects, who did not activate their CIS brain-pull, could not prevent influx of energy into the peripheral energy stores.

**Figure 4:**
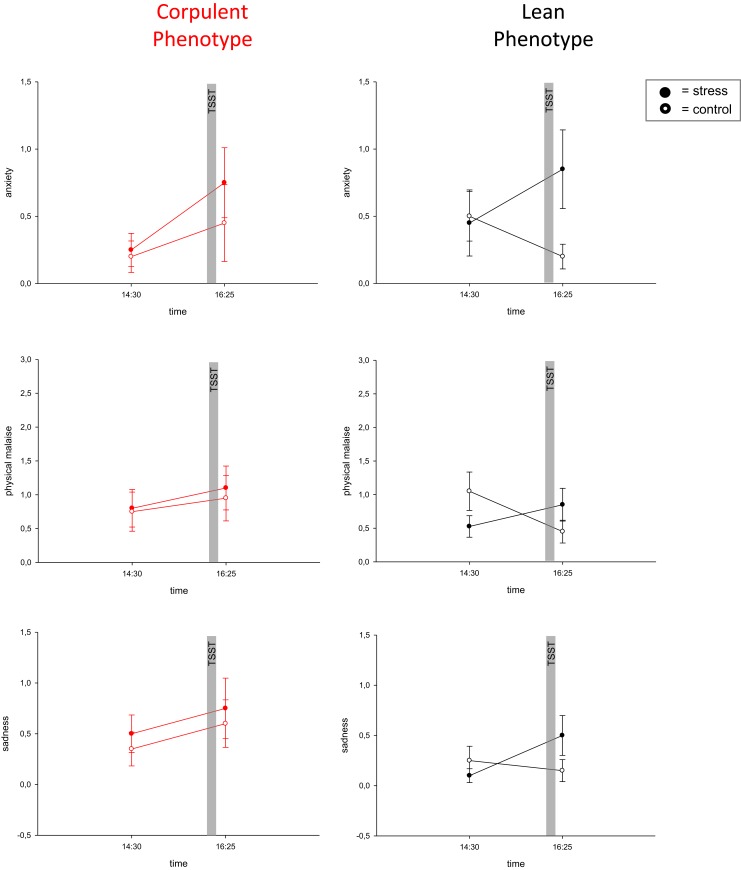
**Blunted emotional arousal during stress in the corpulent phenotype.** Values are means ± SEM; closed symbols, stress intervention and open symbols, non-stress intervention. While lean subjects showed emotional arousal in all symptoms, corpulent subjects remained in their prevailing state of emotion. A*nxiety:* interaction time × stress intervention: *F* = 0.373, *P* = 0.086 (corpulent phenotype), *F* = 11.494, *P* = 0.003 (lean phenotype), *physical malaise:* interaction time × stress intervention: *F* = 0.056, *P* = 0.815 (corpulent phenotype), *F* = 5.979, *P* = 0.025 (lean phenotype), *sadness:* interaction time × stress intervention: *F* = 0.0, *P* = 1.00 (corpulent phenotype), *F* = 5.588, *P* = 0.029 (lean phenotype), by ANOVA for repeated measures (Kubera et al., 2012).

To assess emotional arousal during stress we used a symptom-rating scale (Kubera et al., 2012). The feelings of anxiety, uneasiness, physical malaise and sadness were markedly increased during stress in lean men, but no such emotional arousal could be observed in corpulent men (Figure 4, Table 1).

## The “obesity paradox”—is it due to a confounding factor?

The *association* between high body mass and high mortality risk has been cited very often. However, the assumption that a high body mass is really the *cause* of high mortality has never been tested. This lack of evidence for a causal relationship between the two variables “BMI” and “mortality” is a critical issue, particularly because of the observation that psychosocial stress affects both body mass and mortality (Brotman et al., 2007; Surtees et al., 2008; Steptoe and Kivimaki, 2012). In three large-scale studies, high cortisol concentrations have also been shown to predict increased mortality (Schoorlemmer et al., 2009; Vogelzangs et al., 2010; Kumari et al., 2011). Is it possible that chronic psychosocial stress constitutes one common cause both affecting body mass and mortality? Is it conceivable that psychosocial stress is the key factor that helps to understand the yet unresolved obesity paradox? Recently, Peters and McEwen made a proposal to explain the obesity paradox by introducing stress as the third critical confounding variable (Figure 2B) (Peters and McEwen, 2012).

## Survival advantage in the corpulent phenotype

For decades the BMI-mortality-curve has been used to assess body-weight-related health risks. A BMI of 25 kg/m^2^ has been considered as optimal. Accordingly, each variation in the one direction (weight loss) or in the other (weight gain) shortens life expectancy. Graphically this relation can be depicted as a j-shaped curve. However, in these early statistics the distinction between type-A and type-B-stress types could not be made, because the factor cortisol (allostatic load)—which in the meantime has been shown to have an obvious impact on mortality—has not been assessed.

**Figure 5:**
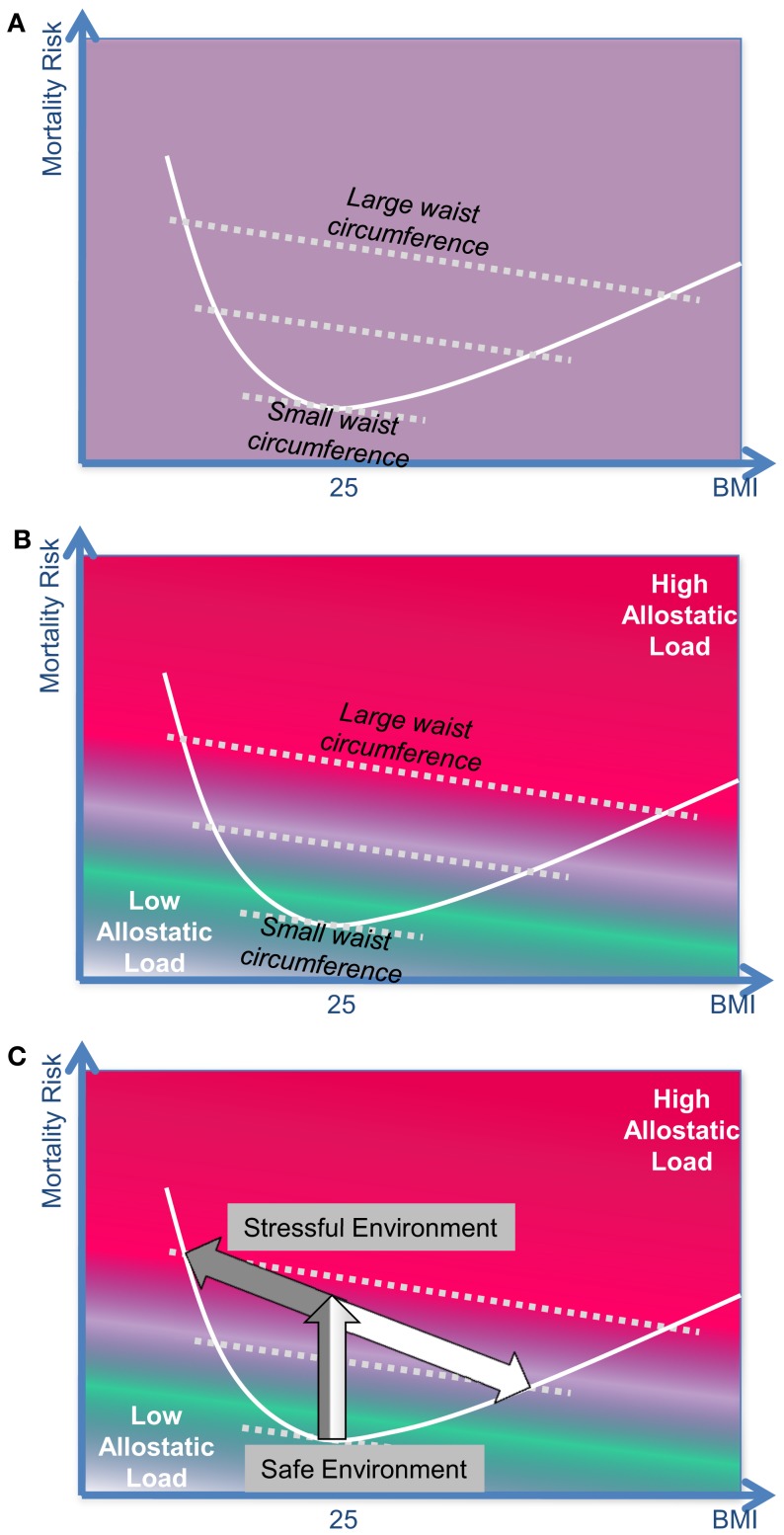
**The BMI-mortality association is confounded by “stress.” (A)** The body mass index (BMI) is not sufficient to predict the mortality risk: modern mortality studies such as the EPIC-STUDY or the DANISH DIET, CANCER, AND HEALTH STUDY take into account both the BMI *and* the waist circumference to predict the mortality risk in humans. **(B)** Waist circumference and allostatic load: the size of the waist circumference can be considered as a clinical marker for the allostatic load a person has carried over the past years. In the upper part of the diagram (red part) the position of those subjects can be found who have carried a substantial stress burden in the past; at the bottom (green part) the position of relaxed, unburdened subjects can be found. **(C)** The effect of stress on the mortality risk in distinct phenotypes: type-A-individuals represent the left arm of the j-shaped curve, while type-B-individuals represent the right arm.

If one does account for cortisol in these analyses, then the following picture can be seen (Figure 5A). The diagram is based on the findings of modern mortality studies like the *EPIC-Study* (Pischon et al., 2008) and the *Danish Diet, Cancer, and Health Study* (Berentzen et al., 2010). These studies do not only rely on the BMI but also on the waist circumference to predict mortality in humans. The diagram-position of most people in a given population can be found close to the j-shaped curve. The diagram-position of individuals who display a particularly large or a particularly small waist circumference can be found in greater distances from the j-shaped curve.

The waist circumference is a clinical estimate of the intra-abdominal (visceral) fat mass. Measurements of the waist circumference can easily be applied in large epidemiological studies. A more exact assessment of the intra-abdominal fat mass requires magnet resonance imaging technique. Recent neuroendocrine studies could show how the intra-abdominal fat grows under the influence of cortisol (i.e., chronic stress). Sympathetic nerve endings projecting to abdominal fat cells release catecholamines and neuropeptide Y; cortisol amplifies the effect of neuropeptide Y acting on visceral fat cells and in so doing leads to hyperplasia in these cell populations (Kuo et al., 2007). The psychiatrist Elissa Epel has emphasized that the waist circumference is a good clinical marker for estimating the allostatic load a subject has been exposed to in the recent decades (Epel et al., 2000). Besides, intra-abdominal fat tissue, which has grown under the influence of cortisol, becomes an almost autonomous organ secreting cytokines, which may further enhance the detrimental effects of the allostatic load. On this background from stress research, it is possible to introduce the confounding factor “stress” into the relationship between “BMI” and “mortality.” In Figure 5B, those subjects who have been exposed to high allostatic load in the recent years find their position in the upper right part of the diagram (red), those who had a low allostatic load find their position in the lower left part of the diagram (green).

How can the BMI-mortality curves be interpreted on the background of **The Selfish Brain Theory?** Our approach distinguishes between the reactions of the two stress types A and B (Figure 5C):

- If type-A-individuals are moved from a safe environment into a stressful and unsafe environment, then their stress system remains high-reactive in the long run, their mortality risk increases (*in the diagram their position moves upwards*), and they lose body weight (*position moves to the left*): These type-A-individuals represent the left arm of the j-shaped mortality-curve.
- If type-B-individuals are set into a stressful and unsafe environment, their mortality risk increases at first (*position moves upwards like in type A*), but then they habituate to the chronic stress, their stress system becomes low-reactive thereby decreasing the allostatic load, and in this way their mortality risk is lessened. However, these subjects have to eat more to procure their brain with sufficient energy, and thereby they gain body weight (*position moves to the lower right*): these type-B-individuals represent the right arm of the j-shaped mortality-curve.

Thus, two distinct phenotypes may develop under stressful conditions, a process which is represented by a bifurcation of the mortality curve (this bifurcation is essentially based on the bifurcation shown in Figure 2A). It becomes obvious that *stressed* corpulent people have a lower mortality risk than *stressed* lean people.

## Revisiting evidences from human mortality studies

There are three classes of clinical studies: those in which the confounding effect of “stress” is unknown, negligibly small, or controlled. The first class of studies is based on the BMI only. There is no information available on the allostatic load experienced by the participants. Thus, it cannot be excluded that *stressed* corpulent people have been compared with *unstressed* lean people. As a consequence of such a mistake, inconsistent results have been obtained within this class of studies. For example, studies on patients with coronary artery disease included both those reporting *negative* associations between the BMI and mortality (Gruberg et al., 2002, 2005; Gurm et al., 2002; Powell et al., 2003; Lopez-Jimenez et al., 2004; Eisenstein et al., 2005; Kennedy et al., 2005; Sierra-Johnson et al., 2005; Nikolsky et al., 2006; Mehta et al., 2007; Lavie et al., 2011) as well as those reporting *positive* associations between these two variables (Rea et al., 2001; Schwann et al., 2001; Kaplan et al., 2002; De Bacquer et al., 2003; Kim et al., 2003; Kuduvalli et al., 2003; Poston et al., 2004; Rana et al., 2004; Wessel et al., 2004; Widlansky et al., 2004; Dagenais et al., 2005; Domanski et al., 2006; Martin et al., 2006; Nigam et al., 2006; Benderly et al., 2010). Such conflicts have ignited a long lasting debate. The uncertainty in interpreting these inappropriate data sets is reflected by the term “obesity paradox.”

The second class of studies is also based on the BMI, but in these studies the confounding effect of the allostatic load is negligibly small. Within this class of studies there are two subgroups. The first subgroup of studies consists of those, which have been carried out in critically ill patients, e.g., those who had suffered from stroke, intracerebral hemorrhage, myocardial infarction, heart failure, end-stage kidney failure, or sepsis. It is likely that the allostatic load was enormously high in all of these cases. Therefore, the variance in the allostatic load was rather small, and the confounding effect of “stress” was negligibly. These studies consistently showed *negative* associations between BMI and mortality (Garrouste-Orgeas et al., 2004; Buettner et al., 2007; Hallin et al., 2007; Fitzgibbons et al., 2009; Kalantar-Zadeh et al., 2010; Kim et al., 2011; Vemmos et al., 2011). The second subgroup of studies consists of those, which have investigated subjects from general populations. Interestingly, when the researchers confined their analysis to BMI-values in the neighborhood of 25 kg/m^2^ (were allostatic load is negligibly small) they also reported *negative* associations between BMI and mortality (Flegal et al., 2013).

The third class of studies controlled for the confounding effect of “stress” by using statistical means (Pischon et al., 2008; Berentzen et al., 2010; Petursson et al., 2011; Cameron et al., 2012; Hamer and Stamatakis, 2012). These researchers used the BMI, but in addition they applied the waist circumference in a multivariate approach. Since the waist circumference serves as a clinical marker of the allostatic load experienced by the participant during the recent decades, they could statistically adjust the BMI-mortality association for the confounding effect of stress. These latter studies consistently demonstrated *negative* associations (negative partial regression coefficients) between BMI and mortality.

As has been already proposed be many researchers and clinicians, the exclusive use of the BMI in mortality studies should be abandoned. Instead the multivariate approach—making use of both the BMI *and* the waist circumference—appears more appropriate.

## The allostatic load is low in the corpulent phenotype

When comparing the health risks of chronically stressed type-A and type-B-individuals we find strong evidence for several differences that are summarized in Table 2: while A-types remain high-reactive in their stress reactivity, B-types habituate and develop a low-reactive stress system (compare also Table 1). A-types lose body weight, but they accumulate visceral fat. In contrast, B-types gain body weight, but do not accumulate much abdominal fat because of the alleviated influence of cortisol. When comparing the clinical traits, which develop under the influence of the allostatic load, we find that A-types display a shortened life expectancy, infertility, arteriosclerosis, hypertension, coronary heart disease, myocardial infarction, stroke, typical depression, muscle atrophy, and osteoporosis. In contrast, B-types—although exposed to a stressful environment—carry a markedly reduced allostatic load because of their low-reactive stress system. Accordingly, the clinical traits due to allostatic load are far less developed in B-types. Therefore, the corpulent phenotype appears to be more stress tolerant.

**Table 2:**
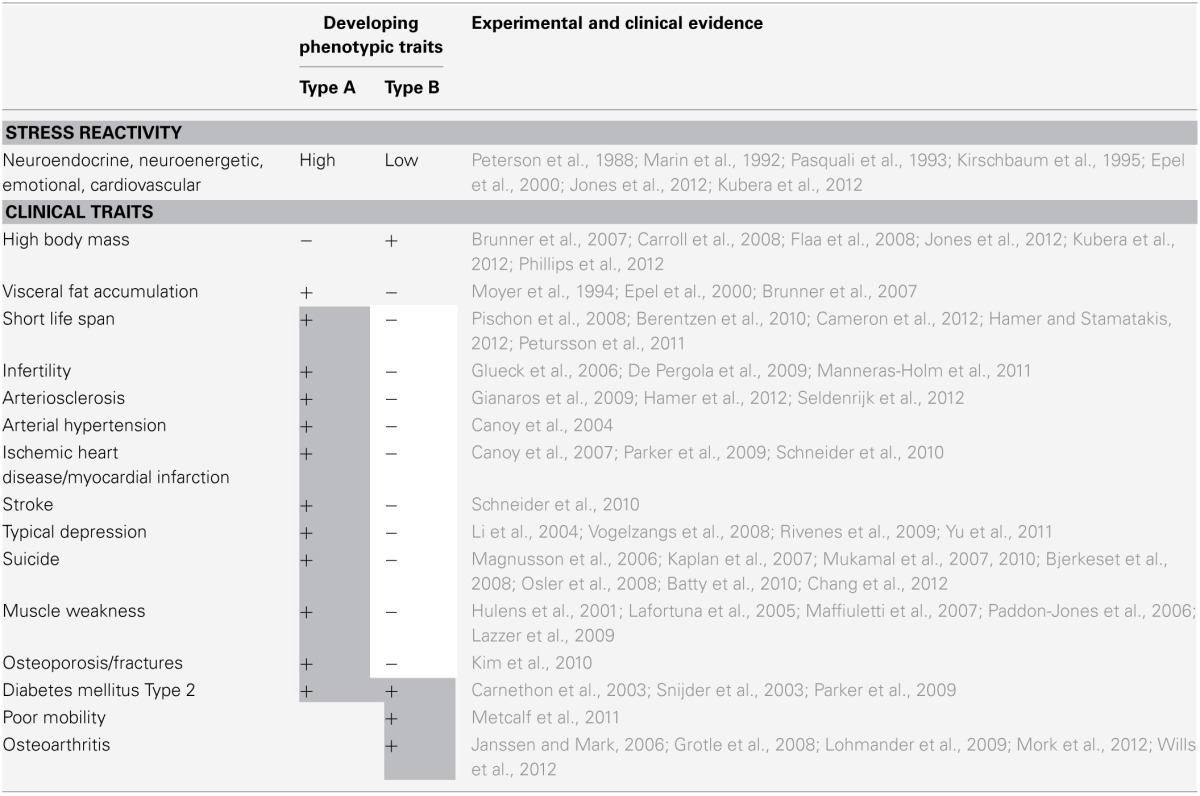
**Distinct phenotypic traits developing in stressful environments**.

Dark gray background indicates “health detriment”; white background indicates “health benefit”; (+) denotes a risk factor promoting the development of the clinical trait; (−) denotes a protective factor suppressing the development of the clinical trait; (not filled) denotes lack of evidence for association.

Noteworthy, type 2 diabetes mellitus can develop both in type A and type B (Carnethon et al., 2003; Snijder et al., 2003; Parker et al., 2009). On the one hand, an increased risk of developing type 2 diabetes mellitus is observed in subjects exhibiting a large waist circumference combined with a low hip circumference—this combination is a characteristic of type-A phenotypes in stressful environment (Snijder et al., 2003; Parker et al., 2009). On the other hand, an increased risk of developing type 2 diabetes mellitus is also observed in subjects exhibiting a low heart rate variability—which is a characteristic of type-B phenotypes in stressful environment (Carnethon et al., 2003). Some years ago, diabetes mellitus type 2 used to be classified by distinguishing a phenotype with low BMI (diabetes mellitus type IIa) and from a phenotype with high BMI (diabetes mellitus type IIb) (Anonymus, 1997). Therefore, this former classification seems to represent the stress types A and B. Thus, the disadvantage of developing type 2 diabetes is not restricted to one particular stress type.

There are also costs which type B has to pay for benefiting from the reduced allostatic load: in order to maintain cerebral energy homeostasis type B has to eat more. In this way brain metabolism is balanced, i.e., cerebral energy concentrations are adequate, and there is neither the need for over activating the stress system (exaggerated energy demand) nor the need for shutting down cerebral function (energy sparing). But as a side effect of this strategy, energy accumulates in the body periphery. The high body weight results in osteoarthritis and impaired physical mobility, both constituting disadvantages for type B.

Table 2 is based on the following specific evidences: it includes longitudinal data sets, in which both the effect of the BMI *and* the effect of the waist circumferences on a certain clinical trait could be assessed. It is crucial that in the data-analyses both the BMI *and* the waist circumferences have been used as independent variables in one multivariate statistical model. Only if this criterion held true, then this data set has been included in Table 2. Because the long-term effects of stress on the respective clinical traits listed cannot be disregarded, the waist circumferences as a clinical marker of allostatic load appears to be useful for a more profound analysis: by controlling for the confounding effect of stress, the waist circumference allows to more precisely evaluate the effect of the BMI (or the hip circumference) on a particular clinical trait.

Two population-based studies from Mauritius and Denmark have analyzed the association between BMI and the mortality risk more profoundly (Berentzen et al., 2010; Cameron et al., 2012). Specifically, the researchers controlled for the confounding effect of waist circumferences (marker of allostatic load). Both studies clearly showed for a wide range of BMI values: the higher the BMI, the lower the mortality risk. One recent British study even showed that corpulent subjects (BMI > 30) without metabolic abnormalities (lack of visceral fat accumulation, lack of hypertension, elevated blood glucose or elevated blood lipids) displayed a life expectancy that was comparable to that of subjects with a “normal” BMI (Hamer and Stamatakis, 2012). Thus, when comparing type A and type B individuals exposed to chronic stress, it turns out that type B individuals do have the better survival chances.

Stress also plays a crucial role in the development of arteriosclerosis (Table 2). Data from the Whitehall Study showed that study participants who were high-reactive in a stress test and who also were burdened by psychosocial stress over the last 15 years had a dramatically increased risk of developing coronary arteriosclerosis (Seldenrijk et al., 2012). Those who were low-reactive and had the same long-term stress burden had a markedly lower risk to develop coronary artery disease. A similar low risk was found in those subgroups of participants who were not exposed to psychosocial stress over the last 15 years. Other studies confirmed the role of stress load and stress reactivity on the development of arteriosclerosis in cerebral vessels. In addition, humans whose amygdala responded in a low-reactive manner to stress stimuli had a markedly reduced risk of developing arteriosclerosis in the large brain supplying arteries (Gianaros et al., 2009).

Recent long-term studies confirm that—when controlling for the confounding effects of stress—high BMI is related to a lower risk of developing arterial hypertension, myocardial infarction and stroke (Table 2). Other studies confirm that the risk of developing a typical depression or the risk of committing suicide is increased in type A (those with an increased abdominal fat mass) while the risk is decreased in type B (high BMI). In the corpulent phenotype, the depression-scores are often found alleviated, and a change in the clinical picture is observed. The corpulent subjects more often present with “atypical depression,” which is not only characterized by increased ingestive behavior, but also by prolonged sleeping time. Muscle atrophy and osteoporosis tend to rather develop in those with a high-reactive stress system when exposed to chronic stress. In contrast, all these clinical traits do not develop in type B individuals to the same extent.

In summary, strong evidence (Table 2) supports the notion that exposure to chronic stress results in two different phenotypes depending on the genetic background. It turns out that type A—which does not habituate to chronic stress—experiences the unbuffered detrimental effects of the allostatic load. In contrast, type B—once exposed to a stressful environment—habituates with his stress response and in so doing is protected against the deleterious effects of the allostatic load. However, this latter kind of adaptation costs energy. The type-B-subjects have to eat more in order to keep their brain energy metabolism in balance. Then the surplus of energy accumulates in the body periphery. At the expense of weight-induced osteoarthritis and reduced physical mobility, type B benefits from a better overall health and longevity in stressful environments.

## Conclusions

Changing the perspective by looking upon the brain as the final consumer in human energy metabolism makes a striking difference: first, it eliminates apparent conflicts that might have occurred during interpretation of clinical data (e.g., “obesity paradox”). Second, “obesity” does not appear to be a disease but rather a phenotypic trait, which represents a successful adaptation in stressful environments. A “corpulent phenotype” can be regarded as the result of adaptive phenotypic plasticity. Third, efforts that aim at safely overcoming corpulence should focus—as exemplarily demonstrated (Ludwig et al., 2011, 2012)—on how people may exit their stressful environment.

### Conflict of interest statement

The authors declare that the research was conducted in the absence of any commercial or financial relationships that could be construed as a potential conflict of interest.

## Key Concept

The Obesity Paradox: The term reflects the controversy which arose from conflicting study results: while some studies reported negative associations between body mass and mortality risk, others reported positive associations between the two variables.
Brain-pull: The brain regulates energy homeostasis via “brain-pull mechanisms,” which function by demanding energy from the body. Two classes of brain-pull mechanisms have been detected so far: first, allocative brain-pull mechanisms, which activate the stress system to favor energy allocation to the brain, and, second, direct astrocytic brain-pull mechanisms, which enhance glucose transport through the blood–brain barrier.
Cerebral insulin suppression (CIS): With cerebral activation of the stress systems, energy—particularly glucose—is allocated to the brain. With activation of the sympathetic nervous system, insulin secretion from the beta cells is suppressed and the insulin-dependent glucose uptake into body periphery becomes limited. As a consequence of CIS, glucose is available via insulin-independent transport across the blood–brain barrier. In this way, CIS can be interpreted as a brain-pull mechanism.
Phenotypic plasticity: Phenotypic plasticity is an environmentally based change in phenotype. Fundamental to the way in which organisms cope with environmental variation, phenotypic plasticity encompasses all types of environmentally induced changes (e.g., morphological, neuroendocrine, behavioral) that may or may not be permanent throughout an individual's lifespan.
Allostatic load: The concept of “allostatic load,” which has been introduced by McEwen and Stellar (1993), is based on the hypothesis that there is a cumulative physiological risk associated with exposure to psychosocial stressors over the life-course. Adaptation in the face of stressful situations and stimuli causes allostatic load and this, over time, leads to diseases.
The supply chain of the brain: With the central nervous system as the final consumer—describes the energy fluxes from the remote environment to the near environment, through the body and finally toward the brain. The brain regulates energy homeostasis via brain-pull mechanisms.
The body-pull: Functions to demand energy from the near environment by initiating ingestive behavior to replenish body stores and blood glucose concentrations. In this connection, mechanisms driven by extracellular cerebral glucose, which is closely related to blood glucose, fulfill the function of exerting ingestive body-pull.
The selfish-brain-theory: Describes the characteristic of the brain to cover its own energy requirements with the highest priority when regulating energy fluxes in the body. The brain behaves selfishly in this respect. The selfish brain theory has been founded and formulated mathematically between 1998 and 2004. Meanwhile, the interdisciplinary Selfish Brain research group, supported by the German Research Foundation, has provided experimental evidence for the validity of the theory's axioms.
